# Association Between Procrastination in Childhood and the Number of Remaining Teeth in Japanese Older Adults

**DOI:** 10.2188/jea.JE20200366

**Published:** 2022-10-05

**Authors:** Moemi Shimamura, Yusuke Matsuyama, Ayako Morita, Takeo Fujiwara

**Affiliations:** Department of Global Health Promotion, Tokyo Medical and Dental University, Tokyo, Japan

**Keywords:** tooth loss, self-control, community-based

## Abstract

**Background:**

Procrastination is associated with stress and unhealthy behaviors. The oral condition reflects the long-term history of an individual’s stress exposure and oral health behaviors; however, empirical studies on the association of procrastination in childhood with remaining teeth in older age are limited. We investigated the association of procrastination in childhood with the number of remaining teeth among community-dwelling older Japanese adults.

**Methods:**

In total, 1,616 community-dwelling senior residents of Wakuya City (Miyagi Prefecture, Japan) who were enrolled in the National Health Plan & the Medical Care System for the Elderly completed a self-administered questionnaire on the number of teeth. Procrastination was measured using a single binary question about timing of holiday homework completion in childhood. The number of remaining teeth was assessed via a questionnaire with response options of ≥20, 10–19, 1–9, and 0 teeth. Ordered logistic regression models with potential confounders (sex, age, maternal education, childhood socioeconomic status [SES], childhood maltreatment, conscientiousness trait) and mediators (adulthood SES, smoking history, alcohol use history) were estimated.

**Results:**

Forty-six percent of participants reported a higher tendency to procrastinate in childhood. The proportions of participants with ≥20, 10–19, 1–9, and 0 teeth were 39.6%, 22.7%, 24.0%, and 13.7%, respectively. After adjusting for all covariates, a higher tendency to procrastinate in childhood was significantly associated with having fewer remaining teeth (odds ratio 1.28; 95% confidence interval, 1.05–1.57).

**Conclusion:**

A higher tendency to procrastinate in childhood was associated with having fewer remaining teeth in later life.

## INTRODUCTION

In Japan, it is estimated that the proportion of older people in the population will continue to increase, reaching 39.9% in 2060, which would be the highest figure globally.^1^ Tooth loss is a significant health problem that affects both oral and general health in an aged society. It is reported that tooth loss in older people is the pre-stage of oral frailty, which leads to oral hypofunction and oral dysfunction, including eating/swallowing and mastication disorder.^2^ Previous studies revealed that the number of remaining teeth in older adults is associated with various diseases and conditions, such as dementia, functional disability, and loss of healthy life expectancy.^3^^,^^4^

Periodontal disease and dental caries are the major causes of tooth loss^5^ and are influenced by oral health-related behaviors, such as tooth brushing, fluoride use, and dental visits.^6^^–^^8^ The number of remaining teeth in older age reflects the history of an individual’s oral health-related behaviors; therefore, one’s self-regulatory skills in childhood may contribute. “A common and pervasive problem characterized by self-regulation difficulties in the form of delaying the start and/or completion of necessary and important tasks”^9^ is called procrastination, and it has been found to link with poor health status, independent of conscientiousness trait.^10^^,^^11^ The procrastination–health model proposed two main pathways that connect procrastination and poor health: 1) procrastination generates stress and 2) procrastinators delay and avoid health-promoting behaviors.^12^^,^^13^ Indeed, an earlier study found that childhood procrastination was associated with at least one tooth loss in adults aged 20–65 years old.^14^ However, there is a scarcity of evidence on whether childhood procrastination is associated with oral health in later life.

Although oral health outcomes have improved in recent decades, the average number of teeth among adults over 85 is 10 and 46% have no teeth.^15^ Tooth loss can be classified into subcategories, such as edentulism (having no teeth), severe tooth loss, and lacking functional dentition (having <20 teeth). The present study investigated the association between procrastination in childhood and the number of remaining teeth measured by four levels of tooth loss in community-dwelling older people in Japan.

## METHODS

### Study design and setting

This cross-sectional study used the data of community-dwelling older people living in Wakuya City, Miyagi Prefecture, Japan. In 2017, self-administered questionnaires were distributed to 3,676 community-dwelling individuals eligible for specific health checkups (40–74 years old) and 2,885 individuals eligible for health checkups for older senior citizens (≥75 years old). Responses were obtained from 1,109 subjects who underwent specific health checkups and 1,001 subjects who underwent health checkups for older senior citizens (response rate, 32.2%). We excluded respondents younger than 65 years (*n* = 388) to investigate the association between procrastination and the number of teeth in older age. Residents of nursing care facilities were also excluded because they might have difficulty completing a retrospective questionnaire about their procrastination tendencies in childhood (*n* = 23). After further excluding respondents with missing information about the number of remaining teeth (*n* = 83), 1,616 individuals were included in the analysis.

### Dependent variable: number of remaining teeth

The number of remaining teeth was investigated using the following question: “*How many remaining teeth do you have? The teeth covered by crowns are counted as your teeth. The total number of remaining teeth of an adult is 32 including wisdom teeth*.” The response options were “*≥20 teeth*,” “*10–19 teeth*,” “*1–9 teeth*,” and “*no teeth*.” The variable was used as an ordered categorical variable, with a larger number indicating fewer remaining teeth in the analysis.

### Exposure: procrastination in childhood

Academic procrastination is a common form of procrastination in children that has displayed moderate correlations with general procrastination.^16^ Thus, procrastination in childhood was investigated in this study using the following single question: “When you were a child, did you usually start working on assignments in the summer vacation as soon as possible?” The answer “No, I started only at the last minute” indicated a greater tendency to procrastinate. Prior studies that employed a similar question as a measurement of procrastination in childhood reported significant associations with health behaviors and health status among adults^14^ and older people.^17^

### Potential confounders and mediators

Previous studies consistently reported associations of procrastination with sex, age, parenting style and conscientiousness,^18^^,^^19^ which are well known to predict the oral health status.^8^^,^^20^^,^^21^ In addition to sex and age, we measured mother’s educational attainment, childhood maltreatment, and socioeconomic status (SES), which are strongly associated with procrastination and oral health via parenting style^22^^–^^24^ and conscientiousness trait; thus, these variables can be potential confounders. Mother’s educational attainment was classified into three levels (college or more, high school, junior high school or less). Childhood maltreatment was measured by asking about the experiences of physical abuse, verbal abuse, neglect, and observation of domestic violence and classified into three groups based on a total number of experiences (ie, 0, 1, and ≥2). Childhood SES was measured by asking the participant “*How was your standard of living when you were 15 years old*?” Five response categories were used to classify participants into high (upper and upper-middle), middle, and low groups (lower-middle and low). In order to differentiate the effect of procrastination from conscientiousness, a personality dimension which is closely associated with trait procrastination and oral health attitudes and behaviors,^25^ we administered the Japanese version of the Ten Item Personality Inventory (TIPI-J).^26^

In addition, we included potential mediators to evaluate the stress and health behavioral pathway of procrastination to the number of remaining teeth in older age. First, we measured adulthood SES as an indicator of exposure to high levels of stress and a potential mediator in the stress path model. Studies have shown that individuals who have a higher tendency to procrastinate are more likely to encounter high levels of stress, such as lower income, shorter duration of employment, increased risk for unemployment, and poor financial management,^27^ which strongly predict poor oral health status.^28^ We asked the respondent’s longest occupation (manual: clerical, sales/service, skilled/labor, agricultural/forestry/fishery workers, or other; non-manual: professional, technical or managerial worker),^29^^,^^30^ and annual household income (<2.5, 2.5–4.9, and ≥5 million Japanese yen [JPY]). Second, we measured two health behaviors as an indicator of maladaptive coping strategies. We asked the participants about their histories of smoking (never smoker, former smoker, current smoker) and alcohol use history (never drinker, former drinker, current drinker).

### Statistical analysis

Ordered logistic regression models were fitted to estimate the odds ratios and 95% confidence intervals for having fewer remaining teeth. The parallel regression assumption was not violated by Brant’s test^31^ (*P* = 0.23). Three models were developed by sequentially adding potential confounders and mediators as follows: Model 1, adjusted for potential confounders (age, sex, mother’s educational attainment, childhood maltreatment, childhood SES, and conscientiousness); Model 2 adjusted for Model 1 variables and adulthood SES (the longest occupation and current household income); and Model 3 further added health behaviors (smoking and alcohol use history).

Missing independent variables were imputed by employing multiple imputations using the chained equation under a missing at random assumption, and 100 imputed datasets were created. As a sensitivity analysis, analysis using dummy variables representing missing information was also performed. All analyses were performed using Stata 14.2 software (Stata, College Station, TX, USA).

## RESULTS

The mean age of the participants was 76.2 (standard deviation, 7.4) years, and 57.7% of the participants were female. Table 1 presents the characteristic of the participants. A higher tendency to procrastinate in childhood was associated with having fewer teeth in older adults. Among participants with a greater tendency to procrastinate, the proportions of subjects with ≥20, 10–19, 1–9, and 0 teeth were 37.4%, 23.2%, 24.3%, and 15.1%, respectively, compared with 43.4%, 22.7%, 21.8%, and 12.0%, among participants with a lower tendency to procrastinate. Older age, lower mother’s educational attainment, few childhood maltreatment, lower childhood SES, lower occupational status, lower annual household income, and current smoking were associated with having fewer remaining teeth.

**Table 1.** Participant demographic characteristics and the number of remaining teeth (*N* = 1,616)

		≥20 teeth	10–19 teeth	1–9 teeth	Edentulous	

		(*n* = 640; 39.6%)	(*n* = 367; 22.7%)	(*n* = 387; 24.0%)	(*n* = 222; 13.7%)	

		*n* (%)	*n* (%)	*n* (%)	*n* (%)	*P*-value
Procrastination	Low	346 (54.1)	181 (49.3)	174 (45.0)	96 (43.2)	<0.001
	High	275 (43.0)	171 (46.6)	179 (46.3)	111 (50.0)	
	Missing	19 (3.0)	15 (4.1)	34 (8.8)	15 (6.8)	
Sex	Female	365 (57.0)	216 (58.9)	224 (57.9)	128 (57.7)	0.936
	Male	274 (42.8)	151 (41.1)	163 (42.1)	94 (42.3)	
	Missing	1 (0.2)	0 (0.0)	0 (0.0)	0 (0.0)	
Age, years	65–69	230 (35.9)	93 (25.3)	57 (14.7)	25 (11.3)	<0.001
	70–74	155 (24.2)	74 (20.2)	47 (12.1)	27 (12.2)	
	75–79	120 (18.8)	83 (22.6)	90 (23.3)	37 (16.7)	
	80–84	95 (14.8)	67 (18.3)	103 (26.6)	52 (23.4)	
	≥85	40 (6.3)	50 (13.6)	90 (23.3)	81 (36.5)	
Mother’s educational attainment	Junior high school or less	392 (61.3)	229 (62.4)	215 (55.6)	138 (62.2)	<0.001
	High school	91 (14.2)	52 (14.2)	32 (8.3)	19 (8.6)	
	College or more	41 (6.4)	21 (5.7)	13 (3.4)	7 (3.2)	
	Missing	116 (18.1)	65 (17.7)	127 (32.8)	58 (26.1)	
Childhood SES	Low	192 (30.0)	99 (27.0)	124 (32.0)	66 (29.7)	<0.001
	Middle	310 (48.4)	189 (51.5)	171 (44.2)	104 (46.9)	
	High	119 (18.6)	66 (18.0)	60 (15.5)	38 (17.1)	
	Missing	19 (3.0)	13 (3.5)	32 (8.3)	14 (6.3)	
Childhood maltreatment	0	479 (74.8)	265 (72.2)	240 (62.0)	136 (61.3)	<0.001
	1	93 (14.5)	59 (16.1)	65 (16.8)	46 (20.7)	
	≥2	26 (4.1)	9 (2.5)	15 (3.9)	7 (3.2)	
	Missing	42 (6.6)	34 (9.3)	67 (17.3)	33 (14.9)	
Longest occupation	Manual	439 (68.6)	232 (63.2)	253 (65.4)	143 (64.4)	<0.001
	Non-manual	112 (17.5)	55 (15.0)	50 (12.9)	19 (8.6)	
	Missing	89 (13.9)	80 (21.8)	84 (21.7)	60 (27.0)	
Annual household income (JPY)	2.5 million	167 (26.1)	120 (32.7)	148 (38.2)	90 (40.5)	<0.001
	2.5 to <5 million	231 (36.1)	119 (32.4)	94 (24.3)	59 (26.6)	
	≥5 million	115 (18.0)	51 (13.9)	45 (11.6)	30 (13.5)	
	Missing	127 (19.8)	77 (21.0)	100 (25.8)	43 (19.4)	
Smoking history	Current smoker	34 (5.3)	27 (7.4)	35 (9.0)	24 (10.8)	0.016
	Former smoker	158 (24.7)	100 (27.3)	85 (22.0)	47 (21.2)	
	Never smoker	431 (67.3)	225 (61.3)	258 (66.7)	138 (62.2)	
	Missing	17 (2.7)	15 (4.1)	9 (2.3)	13 (5.9)	
Alcohol use history	Current drinker	202 (31.6)	105 (28.6)	101 (26.1)	49 (22.1)	0.308
	Former drinker	53 (8.3)	28 (7.6)	28 (7.2)	18 (8.1)	
	Never drinker	370 (57.8)	226 (61.6)	245 (63.3)	149 (67.1)	
	Missing	15 (2.3)	8 (2.2)	13 (3.4)	6 (2.7)	

JPY, Japanese yen; SES, socioeconomic status.

Table 2 presents the results of ordered logistic regression analyses after multiple imputations. A higher tendency to procrastinate was significantly associated with having fewer remaining teeth after adjusting for age, sex, childhood maltreatment, childhood SES, and conscientiousness (model 1: OR 1.34; 95% CI, 1.10–1.64). The association was attenuated after adjusting for the respondent’s adulthood SES, although it remained significant (model 2: OR 1.30; 95% CI, 1.07–1.59). Smoking and alcohol use history explained 6.7% of the association (model 3: OR 1.28; 95% CI, 1.05–1.57). Similar results were obtained by analyses including missing values as dummy variables (eTable 1).

**Table 2.** ORs and 95% CIs for having fewer remaining teeth (*N* = 1,616)^a^

	Crude	Model 1	Model 2	Model 3

	OR (95% CI)	OR (95% CI)	OR (95% CI)	OR (95% CI)
Procrastination				
Low	ref	ref	ref	ref
High	1.28 (1.07, 1.54)	1.34 (1.10, 1.64)	1.30 (1.07, 1.59)	1.28 (1.05, 1.57)
Sex				
Male	ref	ref	ref	ref
Female	1.02 (0.86, 1.23)	0.94 (0.78, 1.14)	0.85 (0.70, 1.05)	1.07 (0.80, 1.44)
Age, years				
65–69	ref	ref	ref	ref
70–74	1.27 (0.96, 1.69)	1.27 (0.95, 1.69)	1.26 (0.94, 1.68)	1.29 (0.97, 1.73)
75–79	2.29 (1.75, 3.01)	2.45 (1.86, 3.23)	2.40 (1.81, 3.16)	2.55 (1.93, 3.38)
80–84	3.32 (2.51, 4.37)	3.55 (2.68, 4.70)	3.43 (2.58, 4.56)	3.73 (2.79, 4.99)
≧85	7.21 (5.35, 9.72)	7.92 (5.84, 10.76)	7.78 (5.71, 10.61)	8.58 (6.25, 11.79)
Mother’s educational attainment				
College or more	ref	ref	ref	ref
High school	1.65 (1.10, 2.51)	1.52 (0.97, 2.39)	1.41 (0.89, 2.22)	1.42 (0.89, 2.24)
Junior high school or less	1.14 (0.71, 1.84)	1.33 (0.81, 2.20)	1.26 (0.76, 2.07)	1.25 (0.76, 2.07)
Childhood SES				
High	ref	ref	ref	ref
Middle	1.06 (0.83, 1.36)	1.10 (0.84, 1.42)	1.07 (0.82, 1.39)	1.07 (0.82, 1.40)
Low	1.14 (0.87, 1.49)	1.00 (0.75, 1.34)	0.97 (0.72, 1.30)	0.99 (0.74, 1.34)
Childhood maltreatment				
0	ref	ref	ref	ref
1	1.40 (1.11, 1.78)	1.24 (0.97, 1.59)	1.20 (0.93, 1.53)	1.21 (0.95, 1.56)
≧2	1.06 (0.65, 1.73)	1.11 (0.68, 1.83)	1.00 (0.61, 1.66)	0.98 (0.59, 1.62)
Conscientiousness	0.86 (0.79, 0.94)	0.84 (0.77, 0.92)	0.85 (0.78, 0.93)	0.85 (0.78, 0.93)
Longest occupation				
Non-manual	ref		ref	ref
Manual	1.42 (1.10, 1.82)		1.35 (1.02, 1.79)	1.34 (1.01, 1.77)
Annual household income (JPY)				
≧5 million	ref		ref	ref
2.5–<5million	1.81 (1.37, 2.40)		1.55 (1.16, 2.06)	1.51 (1.13, 2.01)
<2.5 million	1.05 (0.79, 1.40)		1.07 (0.80, 1.43)	1.07 (0.80, 1.43)
Smoking history				
Never smoker	ref			ref
Former smoker	0.96 (0.78, 1.19)			1.40 (1.03, 1.90)
Current smoker	1.70 (1.21, 2.38)			3.02 (2.03, 4.49)
Alcohol use history				
Never drinker	ref			ref
Former drinker	0.75 (0.61, 0.92)			0.86 (0.66, 1.12)
Current drinker	0.86 (0.62, 1.21)			0.80 (0.54, 1.17)

CI, confidence interval; JPY, Japanese yen; OR, odds ratio; ref, reference; SES, socioeconomic status.

^a^Multiple imputation was applied. Model 1: Sex, age, mother’s educational attainment, childhood SES, childhood maltreatment, and conscientiousness were adjusted.

Model 2: Model 1 + the longest occupation and annual household income were adjusted.

Model 3: Model 2 + smoking history and alcohol use history were adjusted.

## DISCUSSION

This is the first study to investigate the association between procrastination in childhood and the number of remaining teeth in old age. A higher tendency to procrastinate in childhood was significantly associated with fewer remaining teeth after adjusting for potential confounders including conscientiousness. The association was attenuated when we further adjusted for potential mediators (ie, adulthood SES, smoking, alcohol use history), but it remained significant.

Our findings are consistent with earlier studies, which reported a link between general procrastination and elevated stress and lower uptake of health-promoting behaviors among adults^12^ and a link between procrastination in childhood and missing one or more teeth in adults aged 20–65.^14^ Tooth loss is related to dental caries and periodontal diseases, which are affected by lifestyle behaviors, tooth brushing, fluoride use, sugar consumption, smoking, and dental visits.^6^^–^^8^ People with a higher tendency to procrastinate might be at increased risk of periodontal diseases because of high stress levels and maladaptive stress-coping activities, such as sugar intake and smoking. People with a higher tendency to procrastinate may also be less likely to perform highly demanding disciplinary practices with delayed benefits, such as tooth brushing.^14^ Because it is difficult to change a habit once it has been acquired,^32^ the postponement of healthy habits in children may continue unconsciously into adulthood, explaining the fewer number of remaining teeth in older age. In the present study, we investigated the mediating effect of smoking and drinking habits, but the effect of other potential mediators, such as interpersonal stress and other oral health-related behaviors, were not examined. These pathways must be examined in further studies using validated measurements of cumulative life stress and other oral health-related behaviors.

Our study had several limitations. First, this study was a cross-sectional study and the exposure was assessed retrospectively. Missing teeth might have influenced their autobiographical memory recall ability, although our participants were living in communities independently. Childhood procrastination tendencies were measured in a simple form in association with school time, one of the autobiographical memory peaks in life,^33^ and academic behavior has been demonstrated to reflect trait procrastination. However, current procrastination tendencies might have affected the recall of academic procrastination tendencies in childhood. Second, the number of remaining teeth was investigated via self-report rather than clinical examination, although the questionnaire’s validity has been confirmed via clinical examination.^34^ Third, the relatively low response rate might have limited the generalizability of these findings. Fourth, there might be residual confounders, such as impulsivity or a lack of general self-control that are associated with procrastination tendencies in childhood and tooth loss in older age. Fifth, although previous studies suggested maladaptive coping strategies and lower adaptation of health-promoting behavior as potential behavioral pathways linking procrastination and health,^12^^,^^13^ we only measured smoking and drinking history. Further studies measuring oral health-related behaviors, such as sugar consumption, tooth brushing, flossing, and dental healthcare utilization, are needed.

In conclusion, we found that individuals with a higher tendency to procrastinate in childhood had significantly fewer remaining teeth in older age. Further research evaluating the mechanism is needed to better clarify the relationship.

## SUPPLEMENTARY MATERIAL

eTable 1. Odds ratio and 95% confidence intervals for having fewer remaining teeth (N = 1,616)^a^
