# Supplementary material for: Association Between Procrastination in Childhood and the Number of Remaining Teeth in Japanese Older Adults
Source: J Epidemiol. 2022 Oct 5;32(10):464–8. doi: 10.2188/jea.JE20200366 (PMC9424186; doi:10.2188/jea.JE20200366)
Supplement: Supplementary file 1 [file je-32-464-s001.pdf]

eTable 1. Odds ratio and 95% confidence intervals for having fewer remaining teeth (N=1,616)[a]

	Crude	Model 1	Model 2	Model 3
	OR (95% CI)	OR (95% CI)	OR (95% CI)	OR (95% CI)
Procrastination				
Low	ref	ref	ref	ref
High	1.28 (1.07, 1.54)	1.40 (1.13, 1.73)	1.37 (1.10, 1.70)	1.33 (1.00, 1.76)
Sex				
Male	ref	ref	ref	ref
Female	1.02 (0.85, 1.22)	0.94 (0.76, 1.16)	0.85 (0.68, 1.06)	1.10 (0.71, 1.71)
Age, years				
65–69	ref	ref	ref	ref
70–74	1.27 (0.96, 1.69)	1.28 (0.96, 1.73)	1.28 (0.95, 1.72)	1.28 (0.88, 1.85)
75–79	2.29 (1.75, 3.01)	2.36 (1.76, 3.16)	2.44 (1.82, 3.29)	2.29 (1.54, 3.41)
80–84	3.32 (2.51, 4.37)	3.71 (2.73, 5.04)	3.81 (2.79, 5.20)	5.57 (3.60, 8.62)
≥85	7.21 (5.35, 9.72)	8.89 (6.29, 12.57)	9.06 (6.36, 12.90)	9.87 (5.96, 16.34)
Mother’s educational attainment				
College or more	ref	ref	ref	ref
High school	1.12 (0.70, 1.81)	1.51 (0.95, 2.38)	1.40 (0.89, 2.22)	1.19 (0.68, 2.07)
Junior high school or less	1.58 (1.04, 2.41)	1.44 (0.86, 2.41)	1.33 (0.79, 2.23)	1.14 (0.61, 2.13)
Childhood SES				
High	ref	ref	ref	ref
Middle	1.06 (0.83, 1.36)	1.15 (0.87, 1.52)	1.13 (0.85, 1.50)	0.94 (0.65, 1.35)
Low	1.13 (0.87, 1.48)	0.96 (0.70, 1.32)	0.93 (0.67, 1.27)	0.86 (0.56, 1.32)
Childhood maltreatment				
0	ref	ref	ref	ref
1	1.43 (1.12, 1.82)	1.27 (0.97, 1.67)	1.25 (0.95, 1.64)	1.16 (0.81, 1.66)
≥2	1.02 (0.62, 1.67)	1.13 (0.65, 1.98)	1.05 (0.60, 1.84)	0.90 (0.42, 1.92)
Conscientiousness	0.86 (0.79, 0.94)	0.84 (0.77, 0.92)	0.85 (0.78, 0.93)	0.88 (0.77, 0.99)
Longest occupation				
Non-manual	ref		ref	ref
Manual	1.38 (1.06, 1.78)		1.43 (1.06, 1.94)	1.42 (1.01, 2.00)
Annual household income (JPY)				
≥ 5 million	ref		ref	ref
2.5–4.9 million	1.02 (0.77, 1.36)		1.60 (1.18, 2.17)	1.51 (1.03, 2.19)
< 2.5 million	1.85 (1.39, 2.45)		1.14 (0.84, 1.54)	1.02 (0.71, 1.47)
Smoking history				
Never smoker	ref			ref
Former smoker	0.94 (0.76, 1.16)			1.52 (1.00, 2.32)
Current smoker	1.67 (1.19, 2.35)			3.25 (1.95, 5.43)
Alcohol use history				
Never drinker	ref			ref
Former drinker	0.86 (0.61, 1.20)			0.81 (0.56, 1.16)
Current drinker	0.74 (0.60, 0.90)			0.81 (0.48, 1.36)

CI, confidence interval; OR, odds ratio; ref, reference.

Missing values were included as dummy variables

Model 1: Sex, age, mother’s educational attainment, childhood SES, childhood maltreatment, and conscientiousness were adjusted.

Model 2: Model 1 + the longest occupation and annual household income were adjusted.

Model 3: Model 2 + smoking history and alcohol use history were adjusted.
